# Pycnodysostosis Unveiled: Craniofacial and Dental Manifestations in Focus

**DOI:** 10.1155/crid/9477641

**Published:** 2026-06-30

**Authors:** Khaoula M′Rabt, Soufiane Kharraz, Hind Ramdi, Fatima Zaoui, Abdelali Halimi

**Affiliations:** ^1^ Department of Orthodontics and Dentofacial Orthopedics, Faculty of Dentistry, Mohammed V University, Rabat, Morocco, um5.ac.ma; ^2^ Department of Pedodontics, Faculty of Dentistry, Mohammed V University, Rabat, Morocco, um5.ac.ma

**Keywords:** craniofacial and dental features, early diagnosis, genetic disease, orthodontics, pycnodysostosis

## Abstract

Pycnodysostosis refers to an uncommon autosomal recessive developmental anomaly, which results in decreased bone remodeling, manifesting itself in a variety of clinical and radiographic displays. We report two cases involving two sisters aged 12.5 and 8 years old from a consanguineous marriage. Both presented with distinctive manifestations of the syndrome and were observed at the Dental Consultation and Treatment Center of Ibn Sina Hospital in Rabat. They presented pathognomonic features of the disorder, including short stature, beaked nose, brachydactyly, obtuse mandibular gonial angle and acro‐osteolysis of the distal phalanges. This case report is aimed at highlighting these features, in particular the craniofacial and dental characteristics that help in diagnosing this genetic disorder and to emphasize the role of the dentist in this process.

## 1. Introduction

Pycnodysostosis (PD, OMIM #265800), also known as Toulouse‐Lautrec Syndrome, is a lysosomal genetic disease, first identified in 1962 by Maroteaux and Lamy. It represents a rare form of bone dysplasia characterized by diffuse skeletal condensation due to osteoclast dysfunction. Even though its exact prevalence is unknown, it has been estimated at 1–1.7 cases per 1 million individuals, equally distributed among males and females [1, 2]. Although being a remarkably rare genetic syndrome, it is widespread throughout the world, with cases described in different racial groups and populations [1, 3]. According to a study conducted by Rodrigues et al. in 2017, Africa accounts for only 15 of the 173 cases of pycnodysostosis reported in the literature [3]. However, a systematic review carried out in 2024 revealed the presence of 42 cases on this continent among the 179 cases documented [1]. Despite these numbers, there could be an underestimation, given that some cases may not have been published. Therefore, a simple literature search was conducted to identify existing cases in Morocco, including those published and those used for this work. It revealed the presence of at least 20 cases in addition to the two cases we will consider.

An autosomal recessive inheritance pattern underlies this uncommon osteopathy. Consequently, consanguinity increases the risk of its occurrence; among reported cases, 30% come from consanguineous parents [1]. The syndrome arises from a deficiency in Cathepsin K (CTSK), which is an enzyme mainly expressed by osteoclasts and plays a pivotal role in the degradation of bone matrix proteins, particularly Type 1 collagen, osteopontin, and osteonectin [4]. The encoding gene is located on chromosome 1q21. Therefore, its mutation compromises the function of osteoclasts, which become capable of demineralizing bone while being incapable of eliminating the organic matrix [4, 5]. As a result, affected individuals develop an osteosclerotic phenotype, with increased bone density and reduced medullary space, leading to particularly dense and brittle bones [1, 4].

These patients typically present a short‐limbed short stature, brachydactyly (abnormally short digits) with acro‐osteolysis (a resorption of distal phalanges) in presence of dystrophic nails, cranial suture delayed closure, clavicle dysplasia and a distinctive facial appearance, including exophthalmos, prominent nose with a convex nasal bridge, hypoplastic jaws with an obtuse mandibular angle [1, 6, 7].

This paper is aimed at presenting two cases of pycnodysostosis and discussing the clinical and radiological features, particularly in the craniofacial region, of this rare disease, to enable the professional to play a key role in its early screening and diagnosis.

## 2. Case Reports

### 2.1. Case Report 1

A 12‐year‐and‐5‐month‐old female was referred to the dentofacial orthopedics department of the Dental Consultation and Treatment Center (CCTD), Rabat, for “dental crowding and dysfunctional mastication.” Our patient was born to a consanguineous marriage; her parents are first cousins, normal and healthy. No blood relatives were reported with similar characteristics or condition. Furthermore, the mother stated that she had delivered her daughters at term and at normal weight in hospital. Maternal and neonatal history was unremarkable. The patient′s vaccination was up to date. The past medical history revealed no fractures and no hormonal therapy.

Upon general physical examination, the patient presented a short stature, with a standing height of 143 cm and a weight of 46 kg. Her face is slightly asymmetrical with equal facial thirds, faded cheekbones, and distinct nasolabial folds (Figure 1A,B). The profile (Figure 1C) is almost flat, cisfrontal with a beaked nose and a cleared labio‐mental furrow. She presented with a prominent forehead (Figure 1), exophthalmic eyes with no bluish sclera discoloration (Figure 2A). Her digits were broad, tapered, and stubby, with short, flat nails. The patient could not bring her shoulders together, ruling out clavicular aplasia (Figure 2B–D).

**Figure 1:**
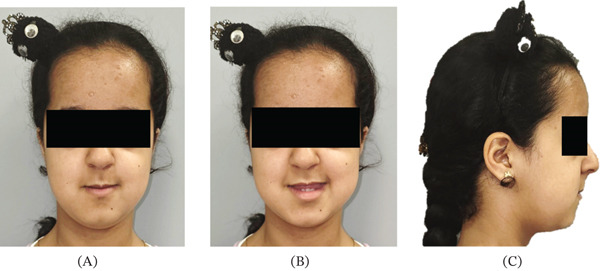
(A) Frontal extraoral photograph, (B) smile photograph, and (C) profile extraoral photograph of the patient, showing a frontal bossing, a beaked nose, and hypoplasia of the middle third of the face.

**Figure 2:**
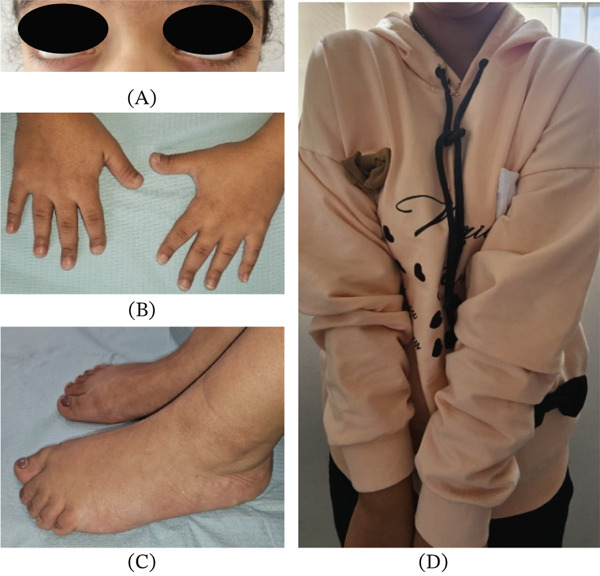
Physical characteristics: (A) Absence of bluish sclera, (B) brachydactyly with characteristic drumstick appearance of terminal phalanges, (C) short toes, and (D) difficulty in bringing the shoulders together.

Intraoral examination (Figure 3) revealed perfectible oral hygiene, in the presence of mild marginal gingival inflammation. Both maxillary arches are hypoplastic. The maxilla is V‐shaped with a deep, narrow, grooved palate. Permanent teeth are present in both arches, but with certain anomalies: The upper left canine is ectopic, delayed eruption of upper second premolars and agenesis of the lower ones, absence of the permanent second molars on the maxillary arch, whereas the mandibular ones are impacted, and the first four permanent molars show occlusal carious lesions, which were subsequently treated. Interarch inspection showed that the maxilla does not circumscribe the mandible, due to its transverse deficiency, and bilateral crossbite involving the posterior and the anterior teeth. The occlusal relationship was Class III, except for the left canine, which was Class II, with a 3–4 mm reverse overjet, a 3 mm overbite as well as a bilateral posterior open bite.

**Figure 3:**
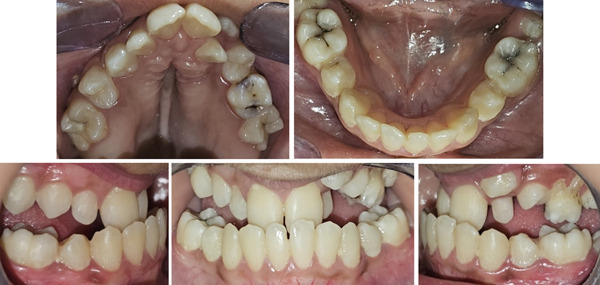
Intraoral photographs of Case 1.

The patient had mixed breathing, combining nasal and oral breathing patterns, dysfunctional swallowing, and mastication. Her mother reported that she snored at night.

Panoramic dental radiograph (Figure 4A) revealed a mandible with a conspicuously thin and narrow body, an obtuse gonial angle, and elongated condylar and coronoid processes. Agenesis of the lower second premolars was confirmed, whereas it was ruled out for the upper permanent second molars; they are impacted rather than congenitally missing. Root length appeared short for all teeth.

**Figure 4:**
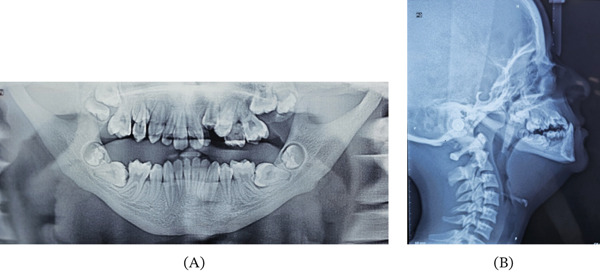
(A) Panoramic radiograph and (B) profile teleradiograph showing obtuse mandibular angle, elongated coronoid and condylar process, Class III skeletal pattern and dental anomalies.

The profile teleradiography (Figure 4B) supported the presence of irregular mandibular morphology. Cephalometric analysis (Table 1) revealed a Class III sagittal jaw relationship concealed by the vertical growth pattern and biretrognathy. The upper incisors were proclined, whereas the lower incisors were retroclined in relation to the mandibular plane. At skeletal age assessment, the patient belonged to the CS‐5 category of cervical vertebral maturation stages [8]. The upper airway space on this X‐ray is narrow, contributing to her mixed breathing and snoring during sleep.

**Table 1:** Cepahalometric values.

Cephalometric measurement	Standard values	Case values
SNA	82^°^ ± 2^°^	70°
SNB	80^°^ ± 2^°^	72°
ANB	2^°^ ± 2^°^	−2°
AoBo	0 ± 2	−8
I to NA	22^°^ ± 2^°^	26°
I to NA	4 mm ± 1	4
i to NB	25^°^ ± 2^°^	18°
i to NB	4 *m* *m* ± 1	6
I to i	131^°^ ± 5^°^	138°
Pog to NB	mm	0
GoGn‐SN	32^°^ ± 5^°^	49°
FMA	25^°^ ± 3^°^	28°
FMIA	67^°^ ± 3^°^	76°
IMPA	88^°^ ± 3^°^	76°
Occlusion plan	10^°^ ± 2^°^	8°
Angle Z	75^°^ ± 5^°^	99°
HFP	45 mm	42
HFA	65 mm	53
Index (P/A)	0.69	0.79

Alongside these X‐rays, the patient was subjected to supplementary radiographic imaging (Figure 5): Frontal teleradiography (Figure 5A) confirmed the enlargement of the skull, the persistence of a slightly open anterior fontanel, hypoplasia of the midface, and showed the presence of a slight mandibular laterognathy, confirming the facial asymmetry noted previously. The chest imaging (Figure 5B) did ascertain the absence of clavicular hypoplasia. The hands and feet X‐rays were truncated (Figure 5D,E), revealing acro‐ostolysis on the thumbs only. The hand and wrist were analyzed to determine a bone age of 15 years, following the Greulich–Pyle method [9]. Bone density is normal, overall, with well‐preserved medullary cavities, particularly those of the long bones of the upper and lower limbs (Figure 5E,F).

**Figure 5:**
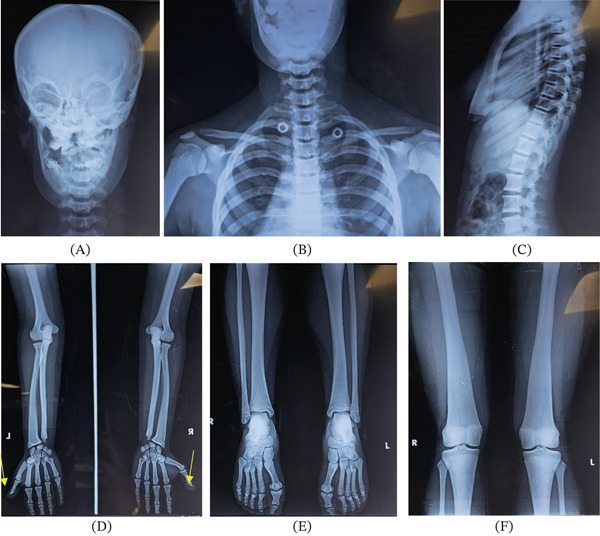
Skeletal X‐rays for diagnosing pycnodysostosis: (A) frontal skull radiograph, (B) clavicular radiograph, (C) lateral spine radiograph, (D) acro‐ostolysis on the distal phalanges, (E) feet radiograph, and (F) bilateral lower limb radiograph.

Given the patient′s family history and her clinical and radiological presentation, a presumptive diagnosis of pycnodysostosis syndrome was made.

Concerning the orthodontic treatment plan envisaged for the patient, as bone remodeling is impaired in these patients, a slow expansion of the maxillary arch using a quad′helix appliance was chosen to ensure molar derotation and expansion. This stage will serve as a therapeutic test to determine the response to dental displacement. The patient is currently undergoing the treatment. If the response is favorable, a camouflage orthodontic treatment will be considered to correct the anterior cross bite, as well as the lateral overbite, to straighten the mandibular second molars and to achieve Class I canine and Class III molar relationships. In fact, the surgical solution seemed the most appropriate to treat this case. However, this decision was postponed due to anatomical and biological constraints, as well as the difficulties presented by the case.

### 2.2. Case Report 2

The following is the second daughter of the same couple, aged 8 years old, who was first diagnosed at the age of 6 years because of her remarkably short stature. She presents a clinically similar picture to that of her elder sister (Figures 6 and 7), whereas the nose appears normal.

**Figure 6:**
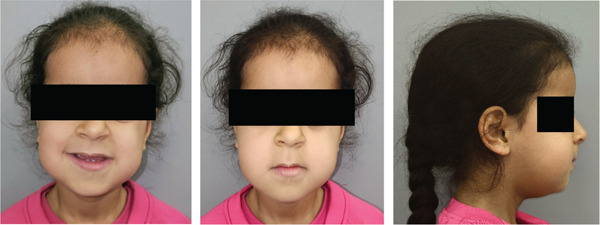
Exobuccal photographs.

**Figure 7:**
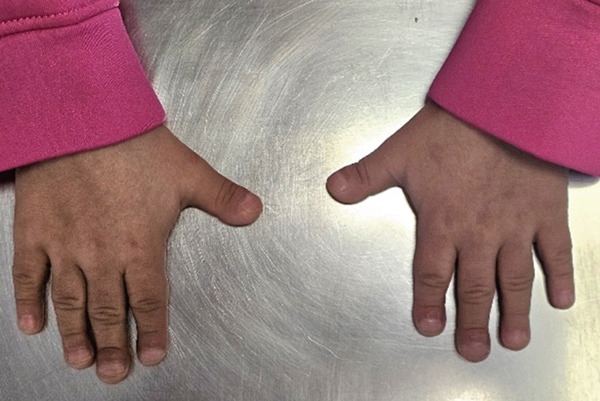
Short hands with drummy fingers′ extremities.

From an oral point of view (Figure 8), localized gingival inflammation was clearly observed opposite the incisor level, as well as a transversely deficient maxilla with a deep median palatal furrow, not circumscribing the mandible, thus leading to a cross‐bite. However, there is a tendency towards an anterior open bite, whereas this is absent in the lateral sectors. The crowding is manifest. It is coupled with dental malpositions, shape anomalies, particularly highlighted in the first mandibular molars, and chronological and quantitative dental abnormalities: premature loss of the first deciduous molars, delayed eruption of the upper permanent laterals, and agenesis of the second premolars. The molars are in occlusal Class III, attributable to the mesial inclination of the first molars, whereas the canines are in Class II because the upper canines occupy the upper laterals site that are lacking on the arch.

**Figure 8:**
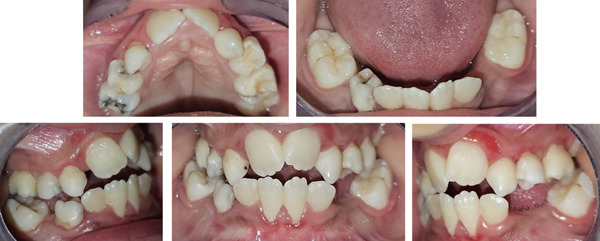
Intraoral photographs of Case 2.

On the panoramic X‐ray and the profile teleradiograph (Figure 9), the shape of the mandible, with its tapering ascending branches and no angle transition to the mandibular body, was also clearly visible. The above occlusal relationships are combined with a hyperdivergent skeletal Class III. Furthermore, on both X‐rays, craniofacial sutures, particularly squamosal, sphenosquamosal, sphenofrontal, and frontozygomatic sutures, are still clearly not closed.

**Figure 9:**
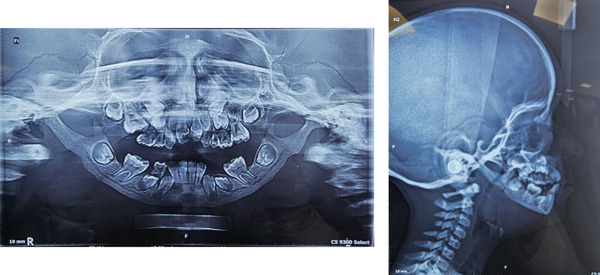
Panoramic radiograph and profile teleradiograph showing the same pattern as her sister.

For the management of this case, after treatment of carious lesions and control of periodontal inflammation, a phase of orthopedic treatment will be considered. The use of a slow expansion appliance is recommended to widen the maxilla and to correct the inverted lateral bite. In addition, correction of the anterior bite, in association with lingual functional rehabilitation, will be undertaken, aiming to strengthen and correct tongue posture and function.

The patients′ approval was obtained, and the parents, as legal guardians, consented in writing to the publication of their child′s case.

## 3. Discussion

Pycnodysostosis is an inherited rare syndrome. Its diagnosis is based primarily on the combination of clinical findings with radiological examination and or genetic investigations [6, 10]. In fact, according to the literature, genetic testing is used to establish a diagnosis—whether through a targeted test or whole‐genome sequencing—only when clinical and radiological findings are inconclusive, particularly when symptoms are mild or atypical [6, 11, 12]. That implies that clinical and radiological features have the primary role in detecting this infrequent syndrome.

The clinical picture of Toulouse‐Lautrec syndrome is varied. It encompasses constant and nonconstant characteristics. Our cases presented short‐limbed short stature, brachydactyly, and craniofacial dysmorphism, which are identified in most occurrences [1, 6]. The short digits may exhibit malformed nails and be covered with wrinkled skin on their backs due to their shortening [6]. Regarding craniofacial dysmorphia, this was particularly evident in a larger‐than‐average head with frontal bossing. Such a distinctive skull shape is caused by a delay in or failure of the cranial sutures to close [10]. Additionally, the eyes are exophthalmic, and the sclera is occasionally colored blue; Bovis et al. [2] and Bizaoui et al. [13] found this to be the case in 40% of the analyzed cases, whereas our patients had a normal sclera. A beaked nose with a high nasal bridge in a retrusive midface is frequent, associated with hypoplastic maxillary bone, as shown in Case 1, compared with a normal nose in Case 2 [3, 6, 7, 11, 13].

Common intraoral traits associated with this disorder included an hypoplastic maxilla with narrow, deep, and grooved palate, mandibular hypoplasia, lateral and/or anterior open bite, cross bite, and various dental malpositions, in particular crowding, impacted teeth, and agenesis, as well as morphologic and structural abnormalities [6, 10, 11, 13]. Our cases are in line with the findings of the literature: The midline groove, a constant feature, appears in our two patients. However, Ferlias et al. [5] reported two children that did not have it. A transverse and vertical deficit of the maxilla has been identified: This deficit leads, respectively, to insufficient space for the eruption of permanent teeth, thus to the retention of deciduous teeth. Reduced vertical alveolar growth in the posterior molar and anterior incisor regions leads to lateral and even anterior open bite. However, in the first case, the anterior open bite is absent; this could be explained by the counter‐clockwise mandibular rotation that occurs when seeking incisal contact [1], whereas in the second case, the opposite is found: A slight anterior open bite is present, whereas laterally, the teeth come into contact, which corresponds to the 29% of cases reported by Bovis et al. [2].

Furthermore, delayed exfoliation of the temporary teeth was observed in the elder sister′s case, only for the upper left temporary second molar, whereas delayed eruption mainly concerned the replacing premolar and the permanent second molars. The latter were blocked as a result of their germs being piled up alongside those of the wisdom teeth in the maxilla and because of their quasi‐horizontal position in the mandible. For the second child, the delay concerned the upper lateral incisors. To some extent, delayed eruption and prolonged retention of primary teeth may be present; however, they are not constant features [1, 6, 14, 15]. In their series, Gonzaga et al. [1] identified tooth agenesis (in 15.6% of cases), impacted teeth (14.5%), and delayed eruption (12.3%). In contrast, Bovis et al. [2] observed different frequencies in their sample (agenesis (55%), persistent temporary tooth (32%), and retained mature tooth (64%)). This discrepancy, as well as our cases, highlights the phenotypic variability described.

A routine imaging assessment in orthodontics discloses a cardinal feature of pycnosdystosis, namely the very particular mandibular configuration characterized by an obtuse mandibular angle without the normal, distinct delimitation of horizontally oriented body and vertically oriented ramus, resembling then to Meckel′s cartilage or to a neonatal mandible [10, 16]. In reality, radiological exams may uncover more: They permit the determination of the absence or delay of cranial sutures fusion, confirm the maxillary and mandibular hypoplasia, and display various teeth abnormalities (e.g., roots dilacerations and shortness, hypodontia…) [7]. Our profile teleradiographs reveal a Class III skeletal pattern that is moderately present in pycnodysostosis cases. It is possibly attributed to maxillary retrognathia [15, 16]. Furthermore, the vertical development is often brachycephalic according to Green and Rowe, though some cases were reported as dolichocephalic, as observed in our patients [14]. Since the lack a well‐defined transition between the mandibular body and ramus results in a very obtuse gonial angle, the dolichocephalic pattern appears more consistent.

In the presence of these clinical and radiological signs, pycnodysostosis should be suspected. However, it may be confused with several hereditary diseases, primarily osteopetrosis and cleidocranial dysostosis, due to their similarities [2, 6, 17]. The distinguishing features are mainly skeletal abnormalities of the skull, mandible, and distal phalanges. The differential diagnosis relies on additional radiological examinations: Hands and feet X‐rays highlight osteosclerosis and acro‐osteolysis of the terminal phalanges, key indicators of pycnodysostosis. Chest X‐rays verify either the existence or absence of collarbones. This condition can then be distinguished from cleidocranial dysplasia based on the above and previously mentioned features, besides the presence of clavicles; its dysplasia is an inconstant parameter, and from osteopetrosis by the absence of hematopoietic alterations, a hallmark finding in osteopetrosis [2, 6, 17]. Based on this approach, a diagnostic hypothesis was proposed for our patient.

Pycnodysostosis is more likely to be detected in early childhood, when children show warning signs of growth retardation and persistent open anterior fontanelles. However, when identification goes unnoticed until advanced age, the diagnosis is then made subsequently to a fracture caused by mild or moderate trauma [17, 18]. Impaired bone remodeling entails increased bone density and sclerosis, leading to bone brittleness and, consequently, predisposing afflicted patients to fractures [10, 17, 19]. Meanwhile, although these signs are subtle or none of these episodes occur, these patients may consult a dentist because of tooth eruption or shedding anomalies, malocclusions, crowding, teeth malpositions, and unaesthetic dental arrangements or impaired oral functions, particularly mastication, as was the case for Patient 1. This implies that dentists, practitioners who are often consulted at an early age, can be the first to detect or suspect its existence. In fact, this case illustrates the key role that dentists can play in screening for rare diseases. Undiagnosed early in life, this uncommon condition may go unnoticed, or its diagnosis may be delayed until the patient′s short stature raises questions, or fractures occur, mainly in adulthood, given the decrease in bone vascularization and the increase in hyperdensification [1, 4, 6, 10, 20]. Dentists therefore need to be aware of the unique dentofacial and general features associated with pycnodysostosis to make an accurate and timely referral, thereby ensuring a prompt and appropriate treatment.

Although the treatment plan is tailored to each patient, the characteristics observed in pycnodysostosis can have a significant impact on orthodontic treatment decisions. In cases of maxillary transverse deficiency—a frequently observed trait—rapid maxillary expansion appears to be the appropriate procedure for correcting it. However, increased bone density limits the skeletal response, making it more difficult, uncertain, and unpredictable: applying significant stress or force to hyperdense bone could result in fracture, potentially leading to osteomyelitis, rather than the desired effect [14]. Moreover, impaired bone remodeling could result in resistance to or a slowing of tooth movement and less predictable reactions. Numerous cases require surgical interventions to address skeletal malformations, but paradoxical bone sclerosis‐fragility renders bone manipulation questionable, as the healing responsiveness is unknown; meanwhile, the risk of osteomyelitis increases significantly with this therapeutic approach [14].

In general, patients with pycnodysostosis exhibit normal cognitive development and life expectancy, since the disease is not progressive [11]. However, complications including bone fractures, dental complications, osteomyelitis, and sleep apnea can potentially lead to significant morbidity, reduced life quality, and consequently a poor prognosis [10, 11]. Hence the importance of early diagnosis and the need for collaboration between the involved healthcare providers, given its complex nature and impact on several systems [6, 10, 13, 15].

## 4. Conclusion

To date, pycnodysostosis remains rarely diagnosed. Affected patients can be identified based on a characteristic clinical phenotype and striking radiographic features, including acro‐osteolysis, osteosclerosis, and obtuse mandibular angle, which are clearly distinctive or associated complications.

However, the dental and craniofacial features associated with this rare genetic disorder, as reported in our two cases, serve as a valuable tool for suspecting its presence and taking appropriate actions for these patients: referral to a specialist for diagnosis and potential management.

This highlights the crucial and essential role that dentists can play in the screening, referral for a specialized diagnosis, and oral care of these patients, thereby contributing to a better quality of life and optimal well‐being. These oral and craniofacial traits therefore require greater awareness on the part of dentists.

## Author Contributions

**Khaoula M′Rabt:** conceptualization, investigation, methodology, data curation, visualization, writing—original draft, and writing–review and editing. **Soufiane Kharraz:** conceptualization, investigation, supervision, and writing—review and editing. **Hind Ramdi:** validation, supervision, and writing—review and editing. **Fatima Zaoui:** validation, supervision, and writing—review and editing. **Abdelali Halimi:** conceptualization, methodology, validation, supervision, and writing—review and editing.

## Funding

No funding was received for this manuscript.

## Disclosure

All authors have read and approved the final version of the manuscript. The corresponding author (K.M.) had full access to all of the data in this study and takes complete responsibility for the integrity of the data and the accuracy of the data analysis.

## Ethics Statement

According to institutional policy, formal ethical approval was not required for this single anonymized case report describing routine clinical care. Written informed consent for publication of clinical details and images was obtained from the patients and their legal guardian.

## Consent

All the patients allowed personal data processing and informed consent was obtained from the legal guardians of all individual participants included in the study.

## Conflicts of Interest

The authors declare no conflicts of interest.

## Data Availability

The data supporting the findings of this case report are available within the article. As the case is still under active treatment, follow‐up data remain limited.
